# Pathways, perspectives and pursuits in polycystic kidney disease

**Published:** 2015-12-13

**Authors:** L. V. K. S. Bhaskar, Ramprasad Elumalai, Soundararajan Periasamy

**Affiliations:** ^1^Sickle Cell Institute Chhattisgarh, Raipur, India; ^2^Department of Nephrology, Sri Ramachandra University, Chennai, India

**Keywords:** Polycystic kidney disease, Cystogenesis, Cyst development, Pathways

## Abstract

Polycystic kidney disease (PKD) is characterized by the growth of numerous cysts in the kidneys. When cysts form in the kidneys, they are filled with fluid. PKD cysts can profoundly enlarge the kidneys while replacing much of the normal structure, resulting in reduced kidney function and leading to kidney failure. Autosomal dominant polycystic kidney disease (ADPKD) is a hereditary disease that occurs in one out of 1000 humans. PKD and its causes are being dissected through studies of human populations and through the use of animal models. Mouse models in particular have made a substantial contribution to our understanding of the gene pathways involved in the pathogenesis and the nature of signaling molecules that act in a tissue-specific manner at critical stages of cyst development. PKD has a number of characteristics that make it uniquely challenging for the development of therapies to slowdown disease progression. This review provides current understanding of the etiopathology, pathways involved and therapeutic targets of PKDs.

**Implication for health policy/practice/research/medical education:**

Polycystic kidney disease (PKD) is characterized by the growth of numerous fluid filled cysts in the kidneys. PKD cysts can profoundly enlarge the kidneys while replacing much of the normal structure, resulting in reduced renal function and leading to end-stage renal disease (ESRD). Given that currently, there are no active effective treatments for PKD, other than renal replacement therapy when indicated, the focus of scientists and clinicians ought for the development of therapies to slowdown disease progression. This review focuses on the current understanding of the pathways and therapeutic targets in PKD.

## Introduction

Polycystic kidney disease (PKD) is described by the growth of numerous cysts in the kidneys. When cysts form in the kidneys, they are filled with fluid. PKD cysts can intensely enlarge the kidneys while replacing much of the normal structure, causing in reduced renal function and leading to end-stage renal disease (ESRD). Autosomal dominant polycystic kidney disease (ADPKD; OMIM 173900) and autosomal recessive polycystic kidney disease (ARPKD; OMIM 263200) are important causes of kidney failure, morbidity and mortality in children and adults of all racial groups worldwide (1). The worldwide prevalence is estimated to be between 1:400 to 1:1000 live births for ADPKD (2) and 1 in 6000 to 1 in 40000 live births for ARPKD (3). Although, ADPKD and ARPKD exhibit variations in inheritance pattern, clinical presentation, typical appearance of the kidneys and global prevalence, both diseases are caused by mutations in proteins located in primary cilia (4,5).

## Materials and Methods

For this review, we used a variety of sources by searching through PubMed, Embase, Scopus and directory of open access journals (DOAJ). The search was performed by using combinations of the following key words and or their equivalents; polycystic kidney disease, cystogenesis, cyst development and pathways.

## Genes in polycystic kidney disease

In ADPKD renal cysts are formed along the full length of the nephron with prevalence to the collecting duct (CD) (6,7) and in ARPKD renal cyst formation is virtually restricted to the CD (6,8,9). Several lines of evidence demonstrate that the mechanical stress arising from variations in tubular flow or tubular composition in CD cells elevate [Ca^2+^]_i_ (10-12)_._ These impaired mechanosensitive [Ca^2+^]^i^ responses, observed for both cultured ADPKD (13) and ARPKD cells (14-16), indicating that the disrupted [Ca^2+^]_I_ signaling plays a fundamental role in cystogenesis. Several studies have provided compelling evidence for the function of polycystin-1 (PC-1) and polycystin-2 (PC-2) proteins. Polycystin-2 can act as a calcium-ion-permeable cation channel, and that polycystin-1 may be involved in regulating/localizing this channel, and play a role in regulating calcium homeostasis. A disruption in intracellular calcium homeostasis seems likely to result in many cellular abnormalities associated with cystogenesis in ADPKD. Further, polycystin 1 and 2 appears to be a member of a superfamily of proteins involved in regulation of renal epithelial cell growth. In the kidney, PC-2 like PC-1 is expressed in all nephron segments, with the possible exception of the thin limbs, but is absent from glomeruli (17).

Thus, ADPKD has two disease loci, PKD1 and PKD2, the loss of both copies disrupts this cascade and causes hyperplasia of a given cell resulting in a cyst. The mutations of PKD1 gene located on 16p13.3 are responsible for 85% of cases, remaining 15% by the mutations of PKD2 gene which is located on the chromosome 4q21–23 (18,19). In elderly patients, the PKD2 mutations are more prevalent than the PKD1. Forty percent of PKD patients were shown the progression of ESRD by the age 63 years have disease linked to PKD2 (20,21). However, among the ADPKD patients the mutations of PKD1 and PKD2 produce the identical renal and extra renal manifestations (22).

ARPKD is a recessive disorder, studies using spontaneously aroused PCK rat model and the method of positional cloning in human ARPKD families indicated that ARPKD is caused by mutations in PKHD1 gene that localized to chromosome region 6p12. The PKHD1 gene encodes the protein fibrocystin or polyductin, which homologous to the proteins of PKD1 and PKD2, has been found in the primary cilium and basal body of renal and bile duct epithelium (23). The PKHD1 has expressed at high levels in the human foetal and adult kidneys, but the lower level expression was detected in the liver and pancreas. So far, more than 300 mutations was documented in PKHD1; among these the truncating mutations exhibits most severe cases, are often leads prenatal or neonatal death (24,25). As majority of mutations being rare variants and one third of these mutations seen in single families, only 10%-20% of ARPKD cases are associated common mutations of PKHD1 gene.

## Pathways in polycystic kidney disease

Although, cellular changes and mechanisms involved in initial stages of cyst formation might differ between ADPKD and ARPKD, the pathogenetic mechanisms become increasingly similar as the disease progresses and involve common pathways (26,27). Once the cysts are formed, further the abnormal cells enforce continuous stress on the surrounding tissues, resulting local injury, which is probably accompanied by synthesis of growth factors and cytokines, triggering additional cyst formation (28). Furthermore, the development and enlargement of cysts in ADPKD requires tubular cell proliferation, extracellular matrix abnormalities and trans-epithelial fluid secretion. Many signaling pathways and transcription factors control the development and growth of polycystic kidneys (29).

Fluid flow-induced bending of the primary cilia causes calcium influx into the cell through PC2 channels, allowing for release of calcium from intracellular stores. Calcium influx into cells modulates the activities of calcium-binding proteins (30,31) that can regulate signal transduction pathways leading to changes in gene expression and the control of cell growth and differentiation (32,33). The reduced calcium caused by mutation of PKD1 or PKD2 can inhibit adenylyl cyclase 6 leading to increased cyclic adenosine monophosphate (cAMP). Cyclic AMP may have a central role in cyst growth by stimulating both fluid secretion and cell proliferation (34,35). It has been demonstrated that cAMP inhibits the proliferation of normal renal epithelial cells. In contrast, cAMP promotes the proliferation of cells derived from PKD patients(34). Low intracellular calcium causes cAMP elevation, which in turn stimulates B-Raf, mitogen-associated/extracellular-regulated kinase (MEK) and extracellular signal-regulated kinase (ERK) in cystic kidney cells but not in normal kidney cells. In normal kidney cells, B-Raf is repressed by Akt (protein kinase B) in a phosphoinositide-3 kinase (PI3K) and calcium-dependent manner, but in cystic kidney and calcium-restricted cells, Akt activity is reduced, allowing for activation of B-Raf by cAMP (36).

Abnormalities in a number of other intra cellular signalling pathways not regulated by Ca^2+^ or cAMP have also been reported. These include mammalian target of rapamycin (mTOR) (37), PI3-kinase (38,39), cystic fibrosis transduction regulator (CFTR) (40), Jak2-STAT1/3 (41), NFAT (nuclear factor of activated T cells) (42), and NF-kB (nuclear factor kappa B) signaling (43).

The phosphoinositide 3 kinase (PI 3-kinase) signaling pathway is an excellent candidate for regulation of epithelial tubule formation. It was demonstrated that PI3-kinase plays an important role in the regulation of kidney tubule branching morphogenesis during development (44). PC-1 can induce resistance to apoptosis through the phosphatidylinositol 3-kinase/Akt signaling pathway (38,39). PC-1 can also favor cell migration by regulating PI3-kinase-dependent cytoskeletal rearrangements (38). Another downstream signaling target that is regulated by changes in PI 3-kinase activity levels is the mammalian target for rapamycin (mTOR). There is overwhelming evidence that the PC-1 controls the mTOR pathway and regulates cell size in a Tsc2-dependent manner, by inhibiting the ERK-mediated phosphorylation of tuberin (45).

Cyst growth in ADPKD involves proliferation of the cyst-lining cells and fluid secretion into the cyst lumen due to transepithelial secretion of chloride subsequent to an increase in cAMP mediated by cystic fibrosis transmembrane conductance regulator (CFTR) (46). This suggests that inhibitors of the CFTR Cl-channel might retard cyst growth. However, expression of CFTR protein in PKD cysts is highly heterogeneous and it is therefore postulated that additional ion channels other than CFTR might also attribute to electrolyte secretion that leads to cyst growth. Calcium-dependent chloride channels, that are activated through stimulation of Gq-coupled purinergic receptors (P2YR), are potential candidates for the mechanisms of the secretion (47). Further, epidermal growth factor (EGF) signaling play a major role in renal electrolyte homeostasis and cyst growth in both ADPKD and ARPKD through cellular proliferation of incompletely differentiated epithelial cells and accumulation of fluid within the cysts (48). Since EGF and its related growth factors regulate the epithelial Na^+^ channel activity, it is possible that ENaC inhibition facilitates cyst formation in PKD (49). A coordinated interaction between ENaC and CFTR was found in disease processes associated with dysfunctional CFTR (50,51). Expression of polycystin-1 activates the JAK-STAT pathway in a process that requires polycystin-2, thus upregulating p21 (waf1) and inducing cell cycle arrest in G0/G1. Mouse embryos lacking Pkd1 have defective STAT1 phosphorylation and p21 (waf1) induction indicating that the polycystin-1/2 complex is to regulate the JAK/STAT pathway. Further, mutations that disrupt polycystin-1/2 binding prevent activation of the JAK-STAT pathway (41).

Further support for PC1 signaling through G-proteins comes from PC1 activation of phospholipase C (PLC) mediated by the G*α*q and the subsequent activation of the calcineurin/nuclear factor of activated T-cells (NFAT) pathway. Signaling through this pathway also connects the function of polycystins as regulators of intracellular calcium levels. Exogenous expression of the PC1 C tail domain results in an increase in calcium level in a reaction requiring PLC *β*. This intracellular increase in calcium leads to the activation of calcineurin, a serine–threonine phosphatase that dephosphorylates NFAT. Activated NFAT translocates to the nucleus and regulates target genes at composite NFAT/AP-1 elements. In addition to the evidence for PC1 mediating NFAT activation, NFAT is co-expressed with PC1 in renal tubular epithelial cells of developing and adult mice, proposing that NFAT and PC1 may work together in a pathway (42). HEK293 cells silenced for *PKHD1* showed a higher PI3K/Akt activity, selective inhibition of PI3K/Akt using LY294002 or wortmannin in these cells increased NF-κB activity (43).

## Therapeutic approaches in polycystic kidney disease

The increased understanding of the molecular mechanisms of PKD has provided a number of targets for therapeutic intervention. Molecular pathogenesis of cystogenesis and cyst progression are the targets of current therapy (Table 1). Many signaling pathways and transcription factors control the cystogenesis and cyst progression of polycystic kidneys. Owing to functional redundancy*,* reciprocal reinforcement and feedback loops these pathways should be considered as a part of network. Cells lacking PC1 and PC2 proteins show decreased levels of calcium that trigger adenylyl cyclase and leading to increased levels of cAMP (75). The central role of cAMP in the pathogenesis of PKD and the ability to hormonally modulate cAMP in a cell-specific manner provide opportunities for such strategies in PKD. It is very important to note that vasopressin V2-receptor and somatostatin SSTR2- receptor signaling utilize cAMP as a second messenger and their signaling increase and decrease cAMP respectively. Hence antagonists of vasopressin V2-receptor (aquaretics) and agonists of somatostatin SSTR2-receptor were used to achieve the desired inhibition of intracellular aAMP. Two important aquaretics OPC-31260 and tolvaptan showed reduction in cAMP levels, slowing cystogenesis, and renal enlargement and dysfunction in murine models (76,77). Further, these aquaretics have been approved for the treatment of ADPKD in Japan (78). The somatostatin analogue, octreotide, was found to be effective in slowing progression in liver and kidney cystic disease in a rat model of PKD. Inhibition of cAMP levels and delayed cyst growth in vitro by octreotide provided further scope for the octreotide in the treatment of PKDs (55). A patient with ADPKD receiving octreotide showed simultaneous reduction in hepatic, kidney and breast cystic volume with preservation of renal function (79). Treatment with octreotide long-acting release in Japanese ADPKD patients showed that Octreotide is safe and effective drug in controlling total kidney volume (TKV) and total liver volume (TLV) (80).

**Table 1:** Pathway targets and therapeutic approaches used in the treatment of PKD

**Treatment target**	**Possible therapy**	**Reference**
Increased apoptosis		
CDK	Roscovitine (Seliciclib, CYC202)	(52,53)
Caspases	Caspase inhibitor (IDN-8050)	(54)
Increased proliferation		
cAMP	Vasopressin V2-receptor antagonists	(55,56)
mTORC1 and 2	mTOR inhibitors	(57,58)
Renin-angiotension system	ACE inhibitors/ARBs	(59-61)
Tyrosine kinases	EGFR tyrosine kinase inhibitor	(62,63)
Transcription factors	PPAR-γ agonist	(64,65)
Abnormal extra cellular matrix metabolism		
Increased collagen expression	PPAR-γ agonist	(66)
Increased metalloproteinases activity	MMP inhibitor (batimastat)	(67)
Abnormal fluid secretion		
cAMP	Vasopressin V2-receptor antagonists	(68,69)
CFTR	Thiazolidinone and glycine hydrazide analogs	(46,70,71)
Cilia		
Calcium influx	TRPC and TRPP2 channel blockers	(72)
Inflammation	TNF-α inhibitors	(73)
Polycystin*-*1/polycystin*-*2	Inhibitors of PI3K (LY294002 and wortmannin)	(74)

Abbreviations: CDK, Cyclin-dependent kinase; cAMP, cyclic adenosine monophosphate; CFTR, cystic fibrosis transduction regulator; PKD, polycystic kidney disease.

As cAMP elevation in cystic kidney cells stimulates B-Raf, MEK and ERK (81), their inhibitors were tested for their action in retarding the progression of PKD (82). Administration of an oral MEK inhibitor, PD184352, inhibited renal cyst enlargement in *pcy* mice by suppressing ERK (83). In contrast to this MEK1/2 inhibitor U0126 had no protective effect in the acute perinatal Pkd1 model of ADPKD (84). Further, PLX5568, a novel selective small molecule inhibitor of Raf kinases attenuated cyst enlargement in vitro and failed to improve kidney function in a rat model of ADPKD (85). Thus, the therapeutic value of blocking *MAPK*/*ERK* signaling pathway in PKD is still controversial.

Inhibition of EGF signaling by a EGFR tyrosine kinase inhibitor (EKI-785) slowed the progression of PKD and reduced mortality in rat model (63). Further, EKI-785 and EKB-569 attenuated the development of PKD in Han:SPRD rats (86). In contrast to this over expression and mislocalization of EGFR are not detected at the apical membrane of cystic cells in PCK rats (87), question the potential therapeutic benefits of EGFR tyrosine kinase inhibitors in treating the PKD.

Previous studies demonstrated an increased mTOR signaling (37) in murine models and human ADPKD, while mTOR inhibitors reverse ADPKD progression (88). Further, sirolimus (rapamycin) prevented aberrant activation of mTOR in epithelial cells lining the cysts and decreased polycystic liver volume, in sirolimus-treated transplant recipient ADPKD patients (89). While in one study with everolimus, cyst volume was blunted, renal functional loss was unchanged at the expense of greater side effects (90). In general previously conducted clinical trials could not demonstrate any clinically relevant effect of mTOR inhibition on cyst growth or renal function. However, a recently published randomized controlled pilot study demonstrated a significant increase in ^125^I-iothalamate (iGFR) in ADPKD patients receiving low-dose rapamycin compared with those receiving standard care, without a significant effect on total kidney volume after 12 months (91). As it is not possible to find a treatment plan that satisfy all desired dose levels simultaneously, some compromise between under dosing the target organ and overdosing the surrounding organs has to be found. Treating ADPKD by combining low dose mTOR inhibitors with non-mTOR based treatments is an effective strategy that would maximize efficiency and prevent adverse side effects (92). A study in *pkd1* mice demonstrated dose dependent effects serolimus on mTOR signalling and showed that the conventional doses in man (blood concentrations ~3 µg l^-1^) are ineffective in slowing cystogenesis (93). Capitalizing on the observation that PCKD cells express high levels of folate receptors, folate-conjugated rapamycin has been used as a novel approach to improve direct drug delivery to renal epithelial cells for effectively limiting cyst growth and reducing side effects (94).

Increased renal vascular resistance exhibited by hypertensive patients is the first demonstration of the involvement of RAAS in modulation of hypertension in ADPKD (95). The RAAS contributes to hypertension in ADPKD, but may also independently accelerate renal cyst growth. Presence of RAAS components (AGT, ACE, ANG II and angiotensin II type I receptor) within cysts and tubules and activation of RAAS during cyst expansion in ADPKD has also been demonstrated (96). Angiotensin II receptor blockers (ARBs) increased renal blood flow in ADPKD, while having an acceptable side effect profile (60,97,98). Several studies in animal models of PKD have shown that ACE inhibitors decreased cyst formation and improved renal function (99,100). The studies on ACE inhibitors in ADPKD are inconclusive because they have used small numbers of patients for shorter periods of time (101-105).

## Conclusion

Till date, the treatment options for PKD have been limited to kidney replacement therapy by dialysis or transplantation. Understanding of molecular mechanisms underlying ADPKD pathogenesis led to the development of pathway-based therapies for the polycystic kidney. At each step in the pathway, a new treatment could be developed, but complete inhibition of PKD progression may require a combination of the various treatments.

## Authors’ contribution

LVKSB: Conception, design, drafting the article and final approval of manuscript. RE and SP: Critical revising of important intellectual content, final approval of manuscript.

## Conflicts of interest

The authors declared no competing interests.

## Ethical considerations

Ethical issues (including plagiarism, data fabrication, double publication) have been completely observed by the authors.

## Funding/Support

None.
